# Forecasting of natural gas consumption in China’s logistics industry based on semi-hierarchical control

**DOI:** 10.1371/journal.pone.0325788

**Published:** 2025-06-10

**Authors:** Chongyan Li, Fuzhong Wang

**Affiliations:** School of Economics and Management, Zhejiang University of Science and Technology, Hangzhou, Zhejiang, China; Vietnam Maritime University, VIET NAM

## Abstract

This paper proposes a research framework based on semi-hierarchical control, analyzes the mechanism of gas instead of oil in China’s logistics industry, and uses several forecasting methods to forecast. The research findings include that: (1) the driving mechanism of substitution of natural gas for gasoline and diesel indicates that natural gas is encouraged by China’s policies and the cost of use is lower, China’s logistics industry will reduce its dependence on gasoline and diesel. (2) By using grey forecasting method, regression trend method and Bass model to forecast natural gas consumption in logistics industry, they show that the forecasting results under different circumstances are helpful for China’s government departments to estimate the consumption trend of natural gas in logistics industry according to different market environments. (3) Based on the reverse feedback mechanism of semi -hierarchical control, combined forecasting methods are established, the hard problem that the combined forecasting coefficients are also solved. Combined forecasting methods are useful complements to meet the forecasting demands of logistics industry’s natural gas consumption, and further improve the forecasting accuracy. (4) According to mean relative error, the error percentages of grey forecasting, regression trend method and Bass model are respectively in 5.373%, 2.9%, and 5.94%, the error percentages of combined forecasting methods are within 2.9%−3.1%, the combined forecasting methods have better forecasting stability.

## 1. Introduction

For a long time, the consumptions of high-carbon energy such as coal, gasoline, diesel and kerosene have occupied a large proportion in China’s energy structure. As a low-carbon and clean energy, natural gas has become the key development direction of clean energy in many countries in the world. In the wave of supply-side structural reform of the energy industry in China under the new normal, it is of great significance to increase the proportion of natural gas consumption. China’s demand for natural gas has grown rapidly in recent years. Since 2016, the government has promoted the “coal to gas” project, under the support of Chinese government policies and the rapid development of natural gas infrastructure, the data from National Bureau of Statistics of China show that China’s natural gas consumption demand increased from 207.81 billion cubic meters in 2016 to 377.30 billion cubic meters in 2021, with an annual growth rate of 12.67 percent. However, the production was only 136.87 billion cubic meters in 2016 and 215.55 billion cubic meters in 2021, far less than the demand for consumption. The share of natural gas in the primary energy consumption structure increased from 6.1% in 2016 to 8.8% in 2021, there was still plenty of room to rise.

Data from the Chinese Statistical Yearbook show that China’s natural gas trade has long been in deficit. Although China’s natural gas production has increased, its external dependence has often exceeded 40%, which fully demonstrates China’s passivity in the natural gas sector – the security of supply is in the hands of others. China lacks natural gas and oil,and relies on imports for them. For example, China’s net imports of natural gas rose from 12.44 billion cubic meters in 2010 to 161.83 billion cubic meters in 2021, while the demand for consumption was 377.30 billion cubic meters in 2021, and net imports accounted for 42.9% at a high level of dependence. It is precisely because of China’s long-term natural gas trade deficit and high foreign dependence, in accordance with China’s “14th Five-Year Plan” Modern Energy System Plan (2021–2025), the idea of “protecting oil and natural gas” was proposed, expressing major concerns about oil and natural gas security. Since China implemented the “carbon peak carbon neutral” strategic program (dual -carbon strategy), the development of the natural gas industry is ushering in an opportunity, and the demand for natural gas in all walks of life is strong. From the statistical data of recent years, in the major industries, the industrial demand for natural gas was the largest, the consumption was only 69.175 billion cubic meters in 2010, the consumption reached 267.825 billion cubic meters in 2021, with the highest annual growth rate of 13.1%. The second annual growth rate was the logistics industry, it increased from 10.67 billion cubic meters in 2010 to 36.631 billion cubic meters in 2021, with an annual growth rate of 11.87%, exceeding the growth rate of residential natural gas consumption in the same period.

The current challenge is that China’s natural gas resources are insufficient, and the logistics industry’s natural gas filling stations and other infrastructure are not perfect. Whether it is the logistics industry, the equipment manufacturing industry such as natural gas powered vehicles and boats, as well as the natural gas filling stations in the industrial chain, they are both optimistic and cautious about the consumption trend of natural gas in the logistics industry. The optimism lies in the implementation of the dual -carbon strategy in China, and natural gas as a clean energy, its low emissions, less pollution and cost-effective, can be widely used in civil and industrial purposes, the application will be more extensive in the future, and the caution is that China’s natural gas consumption is heavily dependent on imports, the natural gas industrial development has inherent deficiencies, it is difficult to form strong support for the development of natural gas applications in the logistics industry. Natural gas has good advantages, especially in the carbon emission reduction today, natural gas shall be paid more attention. Since 2006, China has been the world’s largest carbon emitter. In view of the pressure of carbon emission reduction, the logistics industry acted as a major industry of carbon emissions, whose energy consumption structure should be improved. In this paper, we forecast its natural gas consumption of the logistics industry, and suggest China to increase the use of natural gas and other low-carbon clean energy in the logistics industry, so as to help the development of green logistics and the realization of China’s dual-carbon strategy.

### 1.1. Relevant studies on natural gas pricing reform and marketization

The premise of widespread use of natural gas is its economy, and affordable natural gas price is the key. Chinese scholars have been focusing on the objective and direction of natural gas pricing reform. In the past, the major reform direction was proposed from strict control to deregulation and then to comprehensive marketization. However, due to the great influence of policies, natural gas reform is currently under moderate policy control and moderate decentralization. In order to realize efficient allocation of resources, Liu [1] studied to show that the government would determine the price of urban pipeline natural gas on the basis of monitoring and reviewing the cost of pipeline natural gas, but only one example was listed in Shenyang. Although it has reference value, considering that Shenyang is a northern city, the differences between northern and southern cities were not taken into account, which had certain limitations. Li et al. [2] showed that China’s natural gas management method of “benchmark price + floating range” was insufficient, and suggested that the pricing mechanism in different regions should be changed from government control to oil price linkage or to gas competition. Chen et al. [3] and Xie [4] also showed that a single pricing mechanism, whether linked to oil prices or gas competition, could not avoid all market risks, and they suggested that China should improve the diversification level of imported natural gas pricing, combine trade characteristics and adhere to the principle of “long-term stability” and “short-term flexibility”. As can be seen from the above researches, China’s national conditions are different from other countries, the developments of different regions in China are quite different, and the natural gas price is related to people’s livelihood and business, so the suggestions proposed by various scholars are also different. No matter which natural gas pricing scheme has its advantages and disadvantages, it still will take a long time to perfect the price formation mechanism in China.

### 1.2. Relevant studies on natural gas consumption and economic growth

Natural gas is an important energy. There are many researches on natural gas consumption and economic growth in the academic circles at home and abroad. The consensus is that natural gas, as a low-carbon energy, can replace high-carbon energy and will be widely used in the future, which can have promotion on economic growth. As a matter of fact, natural gas will still play an important role in economic growth, such as Apergis& Payne [5] studied the relationship between natural gas consumption and economic growth based on the panel data of 67 countries during 1992–2005, and the results showed that there was a two-way causal relationship between natural gas consumption and economic growth in both short and long terms. Bianco et al. [6] took the residential market as the research object and showed that natural gas consumption was affected by GDP per capita, natural gas price and Heating Degree Days index (HDD). Among them, HDD index (i.e., climate factor) had the greatest influence. Madreimov [7] studied 53 countries with natural gas exploitation data and consumption data, and found that natural gas consumption had a positive and significant impact on economic development by using OLS regression analysis, statistical studies on random effects, fixed effects and GMM also confirmed the same results.

### 1.3. Relevant studies on natural gas consumption and forecasting

Dong et al. [8] predicted that natural gas consumption will continue to maintain a rapid growth due to the gradual easing of natural gas supply market, the continuous promotion of pipeline network construction and domestic and foreign emission reduction pressure. Li et al. [9] recently found that the level of low-carbon environmental protection in Sichuan needs to be improved, because the increase in the proportion of natural gas consumption would lead to the simultaneous increase in carbon emissions of the industry, and the integrated development of natural gas and new energy would become an inevitable trend. Soldo [10] presented a review of forecasting natural gas consumption, its purpose was to provide analysis and synthesis of published research in this area from beginning to the end of 2010. Dalfard et al. [11], Baldacci et al. [12] had also relevant studies on this field. Zhang & Yang [13] used Bayesian Model Averaging (BMA) to forecast China’s natural gas consumption demand from 2015 to 2020, the BMA method could calculate the posterior model probability and tackle the uncertainty problem of models, which overcame the shortcomings of other existing methods. While Zou and Tang [14] used the Gaussian process regression model to forecast China’s natural gas import volume, they found that the absolute percentage error was less than 5%, and the prediction accuracy was high. Zhang [15] used the seasonal differential autoregressive moving average model (SARIMA model) to forecast and evaluate the apparent consumption of natural gas, so as to obtain the expected total consumption of natural gas market. The prediction accuracy of Zhang [15] was high, but the disadvantage was that the prediction time was short, the forecast was on a monthly basis. Similarly, Zheng and Lan [16] also forecasted the demand for natural gas by month, they used the combined model based on XGboost (eXtreme Gradient Boosting) and Prophet (The Prophet model is a time series prediction model that Facebook opened source in 2017) to forecast the demand for natural gas, in which the predicted value of Prophet had a large deviation, while the comprehensive prediction had a small deviation. Wen et al. [17] used Multi-Fractal Detrended Fluctuation Analysis (MF-DFA) and BorutaShap and other methods to forecast natural gas demand, the BorutaShap algorithm was used to screen feature sequences for the best dimensionality reduction, the newly proposed multi-strategy optimization model HBA-XGBoost (Honey Badger Algorithm-extreme Gradient Boosting) had better prediction accuracy than other comparison models. The prediction accuracy in Wen’s paper was higher, but it was still for the forecast in months. According to Zhen [18], the latest forecasting was that China’s natural gas demand would expect to reach more than 500 billion cubic meters in 2030. A novel nonlinear Bernoulli grey model with hybrid accumulation was proposed [18], in which particle swarm optimization was employed to efficiently determine the optimal nonlinear. In terms of short-term or long –term natural gas consumption forecasting, recent research algorithms are shown in the literature [19–27].

### 1.4. The shortage of natural gas forecasting in China’s logistics industry

At present, few Chinese scholars have paid attention to the forecasting of natural gas for the logistics industry in recent years. In the existing relevant literatures, Chen et al. [28] showed that electricity and natural gas accounted for a small proportion of energy consumption in the logistics industry, with the continuous application of low-carbon technology equipment, low-carbon logistics would have greater development. When analyzing the transportation of LNG (liquefied natural gas), Zhao [29] showed that it included pipeline transportation, ship transportation and cryogenic liquid vehicle transportation, the main factors affecting the transportation distance of LNG pipelines included inlet pressure and delivery capacity, and the efficiency of different transportation pipelines was also different. Regrettably, there is still no data on LNG demand forecasts for China’s logistics industry, but there are some studies at the aspect of forecasting energy use and efficiency in transportation or port, an artificial neural networks (ANN) model was used to predict transport energy efficiency using the European Union data [30]. Machine learning models were used in forecasting electricity demand for port microgrids [31].

Through the above analysis, it is found that the domestic and foreign researches on the logistics industry natural gas consumption and forecasting are still very rare. The research gap can summarized as shown in Table 1.

**Table 1:** Research gaps.

Relevant studies	Research gaps
1. Natural gas pricing reform and marketization 2. Natural gas consumption and economic growth 3. Natural gas consumption and forecasting 4. Natural gas forecasting in China’s logistics industry	(1) There are relatively rare existing studies to use a semi-hierarchical control method and apply it to gas forecasting in logistics industry. (2) Many literatures discuss the forecasting accuracy and deviation of a single forecasting method, and rarely solve the hard problem of coefficient determining and practical operation in the combined method. (3) There are relatively rare studies to control error range by reverse feedback mechanism.

As for the research significance of this paper, in a natural gas trade deficit environment in China, the forecasting value of natural gas in the logistics industry is conducive to the formulation of industrial planning and the construction of gas distributed energy stations, and provides a reference for the manufacturing capacity of natural gas powered vehicles, natural gas powered ships, etc. The reduction amount of carbon emission in the logistics industry can be calculated by the forecasting value of natural gas, which is conducive to the concrete implementation of China’s dual-carbon strategy in the logistics industry.

From the perspective of development trends, the states of the art methodologies in forecasting gas consumption include artificial intelligence and machine learning methods, which have been used in forecasting electricity [31]. So far, no application of these advanced methods in the natural gas consumption forecasting of China’s logistics industry has been found. However, artificial intelligence and machine learning mothods involve a large amount of calculation and are not easy to master, so an easy-to-master methodology will be used. There are a semi-hierarchical control mechanism and three differentiatied forecasting methods (grey, regression, bass method) and their combined methods, their systematic and collaborative jobs ensure the high stability and accuracy of the forecasting.

Our main contributions are as follows:

(1) This paper uses a semi-hierarchical control and apply it to natural gas forecasting in logistics industry. The top-down hierarchical control and inverse feedback mechanism for multi-method are examined.
(2) This paper avoids the possible large deviation of single forecasting method through combined forecasting methods, and more stable and low error forecasting results are obtained.
(3) This paper solves the hard problem of combined forecasting coefficients, which can be calculated by the principle of minimum sum of error squares and can also be used conveniently for practical operation. With the help of inverse feedback mechanism of semi-hierarchical control, the combined forecasting coefficients can be further optimized by the sum of error squares in practice, and the forecasting accuracy can be further improved.

In the following context, firstly, the research framework is proposed, and the mechanism of gas-oil substitution in logistics industry is also studied. Secondly, under semi-hierarchical control, several methods are used to make forecasting and comparative study. All models (grey prediction method, regression trend method and Bass model and their combined models) are time series models, which can be used to predict year data, and there is no choice on other seasonal models or non-time series models. Finally, through the above methods, combined with various analysis results, the conclusions of this study are summarized and some policy implications are proposed.

## 2. Research framework and methods

### 2.1. Control mechanism

**Fig 1:**
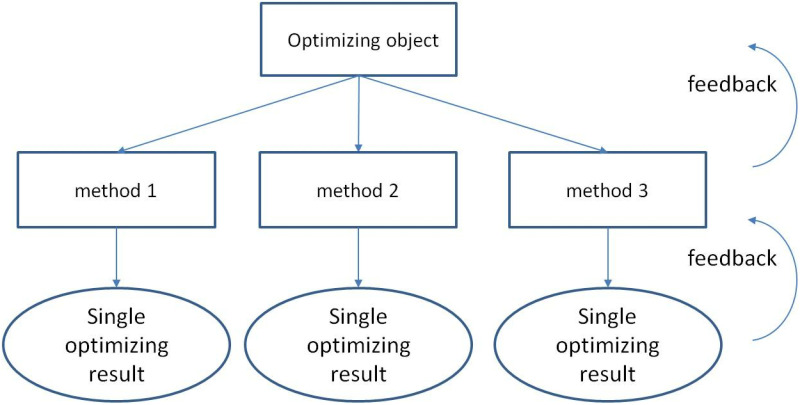
Hierarchical control.

Hierarchical Control was proposed many years ago. Hierarchical control is a kind of master-slave system control. Under the master-slave control mode, the hierarchical control system transmits the control instructions step by step from the top down and the feedback information of command execution step by step from the bottom up. The idea of a hierarchical control mechanism is represented in Fig 1.

As shown in Fig 1, following the top-down problem solving mode of hierarchical control, the complexity of research problems can be reduced and the solving efficiency of problems can be improved. At present, there are relatively rare Chinese scholars have conducted in-depth research on hierarchical control and gradually applied it in the design of logistics system architecture, such as Sun et al. [32] designed a hierarchical intelligent control system of facility agricultural logistics based on AGV(Automated Guided Vehicle), which could optimize the turn times of the path and the number of node traversal in the control process when planning the AGV operation path, and could effectively improve the rationality of the hierarchical scheduling of agricultural logistics. However, there are some studies in energy system via hierarchical control. For example, Hou et al. [33] used Hierarchical model predictive control (HMPC) for energy management and lifespan protection in fuel cell electric vehicles, while Chen et al. [34] used a hierarchical distributed model predictive control (HDMPC) was used to real-time optimal dispatch for large-scale clean energy bases, compared with the existing optimal economic dispatching strategy, simulation results showed that the real-time optimal dispatch via HDMPC can achieve better operational economy, safety and flexibility, etc.

### 2.2. Research framework

**Fig 2:**
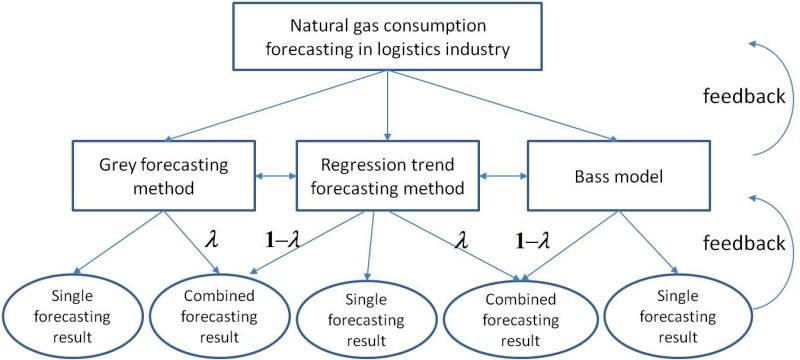
Research framework for natural gas consumption forecasting in the logistics industry based on semi- hierarchical control.

We use a semi- hierarchical control system to pass control instructions step by step from the top down and the feedback information of command execution step by step from the bottom up, and the applicability of each forecasting method can be compared. The research framework is shown in Fig 2.

In Fig 2, the main forecasting methods include grey forecasting, regression trend forecasting, Bass model forecasting and combined forecasting methods. The first three kinds of methods are all single forecasting method, it is hard to meet the forecasting demands of logistics industry’s natural gas consumption. We use the inverse feedback mechanism of semi-hierarchical control, and further make horizontal comparisons for forecasting methods. In forecasting, forecasting methods can also be combined and the combined coefficients (λ) are solved by us. The value of the coefficient (λ) can be obtained by the following the sum of error squares formula:

${Q\;=\;min\Sigma (\lambda *\;method\;1\;forecasting\;value\;+\;(1-\lambda *\;method\;2\;forecasting\;value\;–actual\;value)}^{2}$. (formula Q).

In theory, we use ∂Q/∂λ=0 to solve the optimal λ, which can be solved by programmed processing.

### 2.3. Forecasting methods

#### 2.3.1. Grey forecasting method.

According to Wang [35], the basic idea of grey forecasting is as follows: when there is no obvious trend in a time series, a time series with obvious trend can be generated by the method of accumulation, and the forecasting model can be established according to the growth trend of the latter and the influence of the grey factor can be considered for forecasting.

General modeling is to build difference equations with data columns, while grey modeling is to build differential equations after generating the original data columns, which is generally recorded as GM models [35]. GM (1, N) represents a differential equation type model of order 1 with N variables. For example, the modeling steps of GM (1,1) model are as follows: If the original number is listed as $y^{\left(0\right)}$ and the number generated after one accumulation is listed $y^{\left(1\right)}$, then the differential equation model established according to the cumulative series of generated numbers is $\frac{dy^{\left(1\right)}}{dt}=ay^{\left(1\right)}=u$, and the discrete description form of its solution is as follows [35]:


$$y^{\left(1\right)}(t+1)=(y^{\left(0\right)}(1)-\frac{u}{a})e^{-at}+\frac{u}{a}$$

After the parameter a and u are determined, the cumulative series of forecasting can be obtained by recursion according to this model. After passing the test, the forecasting value can be obtained by reducing again.

#### 2.3.2. Regression trend forecasting method.

According to Wang & Wang [36], the regression trend forecasting is also a commonly used forecasting method. In this paper, the regression model can be set as follows: $\begin{array}{c} Y={\hat{\beta }}_{0}+{\hat{\beta }}_{1}\;T \end{array}$, where T = 1 (2010), T = 2 (2011), then linear regression can be performed according to the original data, and the least square method can be used to obtain:

$\begin{array}{c} {\hat{\beta }}_{0} \end{array}$=$\overset{-}{Y}-\beta_{1}\overset{-}{T}$

$\begin{array}{c} {\hat{\beta }}_{1} \end{array}$=$\frac{\sum \left(T_{t}-\overset{-}{T}\right)(Y_{t}-\overset{-}{Y})}{\sum {\left(T_{t}-\overset{-}{T}\right)}^{2}}$

At this point, the optimal regression equation is determined, and then the regression trend forecasting method can also be used.

#### 2.3.3. Forecasting method of Bass model.

Accordint to Zhu et al. [37], Bass model is a widely used method, the formula is as follows:


$$N_{t}=N*(1-e^{-(p+t)t})/(1+(r/p)*e^{-(p+t)t})$$

In order to maintain the consistency of the symbols, we assume $N_{t}=Y_{t}$, then $Y_{t}$ can be denoted as:


$$Y_{t}=N*(1-e^{-(p+t)t})/(1+(r/p)*e^{-(p+t)t})$$

**Fig 3:**
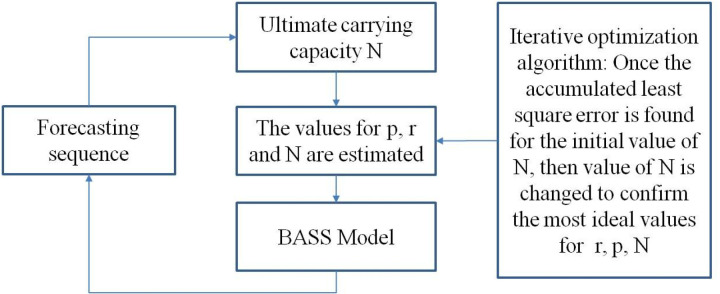
The forecasting method of Bass model.

Here N is the ultimate carrying capacity, *p* is the coefficient of innovation, *r* is the coefficient of imitation, and $Y_{t}$ is the diffusion number at time *t* [37]. The values for *p*, *r* and N are estimated via up-to-now actual diffusion trend [37], which can be estimated by *t*he following ways. The forecasting method of Bass model is shown in Fig 3.

In the iterative process,the decimal part of p and r are limited to three digits.The computational complexity is O(1000*1000), which ensures the accuracy and reliability of the model forecasting.

## 3. Data and gas instead of oil mechanism

### 3.1. Natural gas consumption in logistics industry

The current situation of natural gas consumption in the logistics industry is shown in Table 2.

**Table 2:** Current situation of natural gas consumption in logistics industry.

Year	Energy consumption in logistics industry (Ten thousand tons of standard coal)	Natural gas consumption in logistics industry (Ten thousand tons of standard coal)	Column three/Column two (%)	Natural gas consumption (hundred million cubic meters)	Natural gas consumption in logistics industry (hundred million cubic meters)	Column six/Column five (%)
2010	27102.07	1296.41	4.78	1080.24	106.70	9.88
2011	29694.17	1680.95	5.66	1341.07	138.35	10.32
2012	32560.60	1877.30	5.77	1497.00	154.51	10.32
2013	34819.02	2135.73	6.13	1705.37	175.78	10.31
2014	36343.00	2605.20	7.17	1870.63	214.42	11.46
2015	38510.00	2887.08	7.50	1931.75	237.62	12.30
2016	39883.00	3095.46	7.76	2078.06	254.77	12.26
2017	42140.00	3459.23	8.21	2393.69	284.71	11.89
2018	43617.00	3477.21	7.97	2817.09	286.19	10.16
2019	43909.00	4148.98	9.45	3059.68	341.48	11.16
2020	41309.00	4304.62	10.42	3339.89	354.29	10.61
2021	43935.00	4450.67	10.13	3772.96	366.31	9.71

Data source: China Energy Statistical Yearbook, the unit of natural gas consumption in logistics industry is converted by energy conversion coefficient. The conversion coefficient between 100 million cubic meters and 10 thousand tons of standard coal is 11 ~ 13.3, and the median value is used in this paper is 12.15. In China, transport, storage and post industry are generally referred as the logistics industry.

In Table 1, the natural gas consumption of the logistics industry had been becoming larger and larger, and also maintained a rapid growth speed, with an annual growth rate of 11.87% during 2010–2021. The proportion of natural gas consumption in the total energy consumption of the logistics industry increased from 4.78% in 2010 to 10.13% in 2021, which indicated that the clean power supply represented by natural gas became important.

The rapid growth of natural gas consumption in the logistics industry is a good thing, which also benefits from the promotion and application of trucks and transport ships that use natural gas as energy in the logistics industry, which shows that the logistics industry is gradually green and low carbon. To better understand natural gas consumption growth, we analyze some constructions of natural gas industry. During the 13th Five-Year Plan period (2016–2020) in China, due to the China’s central government’s emphasis on haze control and carbon emission reduction, the constructions of natural gas distributed energy stations opened the curtain. Natural gas distributed energy is different from traditional high-carbon energy, it refers to the use of natural gas as raw material, through the cold, heat and electricity triple supply and other ways to achieve the cascade utilization of energy. Comprehensive energy utilization efficiency exceeds more than 70%, with higher energy efficiency, clean environmental protection, good safety, peak cutting and valley filling, good economic efficiency, natural gas distributed energy is an important development direction of future energy. At present, there are not many natural gas distributed energy stations that have been built in China, and the industry is still in the early stage of growth with great imagination. These natural gas distributed energy stations (including liquefied natural gas filling stations) can provide convenience for the continuous filling work of natural gas powered trucks and ships, and effectively promote the application of natural gas in the logistics industry. Based on economic and environmental reasons, not only the applications of liquefied natural gas (LNG) trucks in northern and southern regions are increasing, but also natural gas-powered ships and other natural gas-powered systems are also beginning to increase, and we expect that in the future, natural gas will have a more important position in the energy consumption structure of the logistics industry.

### 3.2. Mechanism of gas instead of oil

In the last century, fossil energy occupied an absolute leading position in the entire energy system, steam engine trains powered by coal were once an important means of logistics transport, and trucks and internal combustion engine locomotives powered by diesel are still important means of transport at the present. However, in the past ten years, motor driven vehicles and natural gas-driven vehicles and ships have undertaken part of the logistics and transportation tasks. In order to implement carbon neutrality in 2060,China has paid great attention to environmental protection. Natural gas is a kind of clean energy,so the natural gas-powered logistics equipments and transportation tools began to become particularly important.

The logistics industry consumes a variety of energy sources, such as coal, gasoline, diesel, natural gas, electricity, etc. It is assumed that E represents the comprehensive index of energy consumption in the logistics industry, and that E utility is a CES type and E is also a sub-utility function on the continuous space of energy types [38]. The price of each energy is $p_{i}$, the quantity of each energy is $q_{i}$, ρ indicates the consumption preference. If the logistics industry is required to have the lowest cost of energy mix, it requires:


$$\begin{array}{c} \min\int_{0}^{n}p_{i}q_{i}di\\ s.t.\\ \begin{array}{cc} {}*20c & [\int_{0}^{n}{q_{i}}^{\rho }di \end{array}{]}^{1/\rho }=E \end{array}$$ (1)

Because of the substitutability between sub-energy sources, the first-order condition for minimization is:


$$\frac{{q_{i}}^{\rho -1}}{{q_{j}}^{\rho -1}}=\frac{p_{i}}{p_{j}}$$ (2)

For any group of $q_{i}$ and $q_{j}$, there is:


$$q_{i}=q_{j}(p_{j}/p_{i}{)}^{1/(1-\rho )}$$ (3)

We put the formula (3) into $E=[\int_{0}^{n}{q_{i}}^{\rho }di{]}^{1/\rho }$, there is:


$$q_{j}=\frac{{p_{j}}^{1/(\rho -1)}}{{\left[\int_{0}^{n}{p_{i}}^{\rho /(\rho -1)}di\right]}^{1/\rho }}E$$ (4)

Formula (4) is the relationship between the consumption of the energy j and all energy consumption [38]. Combined with the actual situation in China, from the perspective of the evolution trend of gas-oil substitution, it is inevitable that low-carbon clean energy (such as natural gas) will slowly replace high-carbon energy (such as gasoline and diesel). In reality, natural gas-powered trucks have partially replaced diesel heavy trucks, and natural gas-powered ships are slowly replacing diesel powered ships. We take natural gas and diesel in the logistics industry as an example to analyze gas-oil substitution, and write the consumption quantity of these two kinds of energy respectively as $q_{ng}$, $q_{d}$, and the price respectively can be expressed as $p_{ng}$, $p_{d}$. According to equation (3), the function of natural gas replacing diesel can be expressed as:


$$q_{d}=q_{ng}(p_{ng}/p_{d}{)}^{1/(1-\rho )}$$ (5)

According to equation (5), during the period t, equation (6) can be expressed as:


$$p_{ng}^{1/(1-\rho )}q_{ng}(t)=p_{d}^{1/(1-\rho )}q_{d}(t)$$ (6)

We assume that the different input costs of natural gas, diesel and gasoline equipments are respectively $C_{ng}$, $C_{d}$ and $C_{g}$, for natural gas equipments with the same working capacity, taking into account the intrinsic value of science and technology, it should be $C_{ng}$>$C_{d}$ and $C_{ng}$>$C_{g}$, for example, the purchasing cost of the same tonnage of natural gas powered heavy trucks is greater than that of diesel heavy trucks. During the period t, in order to make the logistics industry has an incentive to use natural gas technology equipments, then


$$C_{ng}+p_{ng}^{1/(1-\rho )}q_{ng}(t)<C_{d}+p_{d}^{1/(1-\rho )}q_{d}(t)$$ (7)

Similarly, for gasoline price $p_{g}^{1/(1-\rho )}$ and its consumption $q_{g}(t)$ during the period t, the following formula should also be established:


$$C_{ng}+p_{ng}^{1/(1-\rho )}q_{ng}(t)=C_{g}+p_{g}^{1/(1-\rho )}q_{g}(t)$$ (8)

Even if $C_{ng}$<=$C_{d}$ or $C_{ng}$<=$C_{g}$, the Formula (7) and (8) are also right, the Formula (7) and (8) show that in the period t, the energy cost of natural gas technology and equipment should be less than the energy cost of gasoline or diesel technology and equipment, only in this way, the cost advantage will drive the logistics industry to accelerate the use of natural gas technology and equipment to replace gasoline or diesel technology and equipment.

We take an example to explain the replacement relationship. Hangzhou Tangs Logistics Company, as a representative logistics enterprise in the food and beverage industry, began to use LNG trucks in 2013 to reduce carbon emissions. The company purchased 20 LNG trucks at one time, which increased the cost by nearly 2.4 million yuan compared with diesel trucks. Calculated according to the annual operation time of 350 days, the annual fuel cost could be saved about 76,000 yuan, that is, the increased cost of trucks’ purchasing could be recovered after a year and a half of operation. According to the above data, $C_{nc}-C_{d}$=120000 RMB, daily variable costs$v(day)=p_{d}^{1/(1-\rho )}q_{d}(day)-q_{ng}(day)p_{ng}^{1/(1-\rho )}=218RMB$, annual variable cost v(year)=76000RMB, v(t)*t=120000RMB, when t >=1.5 years, formula (7) is reliable. That is, through a year and a half of comparison, the economy of natural gas-powered heavy trucks instead of diesel heavy trucks will be reflected. This shows that if the time cycle is extended, the economy of natural gas-powered heavy trucks is more obvious.

## 4. Forecasting results

### 4.1. Forecasting results of grey forecasting method

At first, we use the grey forecasting method to forecast. In view of the small consumption of natural gas in the early stage in the logistics industry, the data of the past five years are used as the original series of forecasting in the method, and the forecasting results are shown in Table 3.

**Table 3:** The forecasting results of grey forecasting method.

Year	Forecasting results of grey forecasting method (hundred million cubic meters) t = 5
2010	–
2011	–
2012	–
2013	–
2014	–
2015	243.89
2016	277.96
2017	291.71
2018	310.32
2019	311.98
2020	364.18
2021	388.49
2022	403.23
2023	418.92
2024	446.85
2025	476.78
2026	502.46
2027	535.45
2028	567.55
2029	601.42
2030	638.75
2031	676.79
2032	717.72
2033	742.15
2034	787.72
2035	824.75

Under this forecasting results, the rises of the forecasting values are still large. In order to continue the comparison with other methods, the regression trend method is continued to make the forecasting.

### 4.2. Forecasting results of regression trend method

According to the characteristics of the original data, the unary linear regression model can be used for forecasting, and it is obtained that: , all regression tests are significant, wherein Adj R^2^ = 0.989, the forecasting results by regression trend method are shown in Table 4.

**Table 4:** Forecasting results of regression trend method.

Year	Forecasting results of regression trend method (hundred million cubic meters)
2010	110.47
2011	134.55
2012	158.63
2013	182.72
2014	206.80
2015	230.89
2016	254.97
2017	279.05
2018	303.14
2019	327.22
2020	351.31
2021	375.39
2022	399.47
2023	423.56
2024	447.64
2025	471.73
2026	495.81
2027	519.89
2028	543.98
2029	568.06
2030	592.15
2031	616.23
2032	640.31
2033	664.40
2034	688.48
2035	712.57

The increases of the forecasting value in the regression trend method are smaller than that of the grey forecasting method. Before the comparison of various methods, we continue to use the Bass model for further forecasting.

### 4.3. Forecasting results of Bass model method

By the Bass model, the forecasting results are shown in the following Table 5.

**Table 5:** Forecasting results of Bass model method (unit: hundred million cubic meters).

Year	Bass model forecasting results (1) (*N* = 655.2478, the optimal p = 0.027, r = 0.125)	Bass model forecasting results (2) (*N* = 1156.063, the optimal p = 0.013, r = 0.107)
2010	84.52	88.71
2011	145.81	151.91
2012	166.53	171.44
2013	186.12	189.73
2014	207.02	209.54
2015	229.12	230.92
2016	252.26	253.90
2017	276.21	278.47
2018	300.74	304.62
2019	325.58	332.29
2020	350.44	361.41
2021	375.04	391.87
2022	399.11	423.53
2023	422.39	456.22
2024	444.68	489.75
2025	465.78	523.89
2026	485.58	558.42
2027	503.98	593.09
2028	520.94	627.66
2029	536.45	661.86
2030	550.52	695.48
2031	563.22	728.29
2032	574.60	760.10
2033	584.75	790.72
2034	593.75	820.03
2035	601.72	847.90

In the Bass model of forecasting method, we give two groups of results under different ultimate carrying capacity, in which the optimal parameters of p and r are obtained by repeated iteration. If the annual growth rate (7.53%) in the past five years is used to estimate the ultimate carrying capacity in 2035 (*N* = 65.52478 billion cubic meters), then the Bass model method forecasts that the annual consumption of natural gas in the logistics industry in 2035 will reach 60.172 billion cubic meters. If natural gas consumption in the logistics industry would grow at an annual growth rate of 10% after 2022, the ultimate carrying capacity *N* could reach 115.6063 billion cubic meters in 2035. Then, it is forecasted by Bass model method that the annual consumption of natural gas in the logistics industry in 2035 can reach 84.79 billion cubic meters. These results show that Bass model method has good forecasting results under different expectations.

### 4.4 Combined forecasting results

#### 4.4.1 Combined of grey forecasting and regression trend forecasting.

The inverse feedback mechanism of semi-hierarchical control shows that combinatorial forecasting technology can be used when one or several forecasting results are far correlated with the actual values. The combined forecasting process of grey forecasting and regression trend method is as follows: the value of the coefficient (λ) can be obtained by the sum of error squares (formula Q in the part of research framework). We obtain the optimal λ = 0, the combined forecasting results are shown in Table 6.

**Table 6:** Combined forecasting results of grey forecasting and regression trend forecasting (unit: hundred million cubic meters).

Year	Grey forecasting value*0 + regression trend forecasting value *1
2022	399.47
2023	423.56
2024	447.64
2025	471.73
2026	495.81
2027	519.89
2028	543.98
2029	568.06
2030	592.15
2031	616.23
2032	640.31
2033	664.40
2034	688.48
2035	712.57

Why are the combined forecasting values consistent with the regression trend forecasting values? the reasons are that the original data from 2010 to 2021 are closer to the regression trend value and farther away from the grey forecasting values, so we set the optimal λ = 0, and the optimal forecasting value are the same as that of regression trend method.

#### 4.4.2 Combined of regression trend forecasting and Bass model.

We further use regression trend and Bass model as a combined forecasting, due to setting two different N values, so the forecasting values in Bass model have also two columns, the combined forecasting coefficient (λ) needs to be set twice. The optimal value (λ) for one time is 0.9151 (N = 655.2478), and the optimal value for the other time is 1 (*N* = 1156.063). The forecasting results are as follows in Table 7.

**Table 7:** Combined forecasting results of regression trend and Bass model forecasting (unit: hundred million cubic meters).

Year	Regression trend forecasting value *0.9151 + Bass model forecasting value (1)*0.0849	Regression trend forecasting value *1 + Bass model forecasting value (2)*0
2022	399.44	399.47
2023	423.46	423.56
2024	447.39	447.64
2025	471.22	471.73
2026	494.94	495.81
2027	518.54	519.89
2028	542.02	543.98
2029	565.38	568.06
2030	588.61	592.15
2031	611.73	616.23
2032	634.73	640.31
2033	657.63	664.40
2034	680.44	688.48
2035	703.15	712.57

### 4.5. Comparison and discussion of various forecasting methods

**Fig 4:**
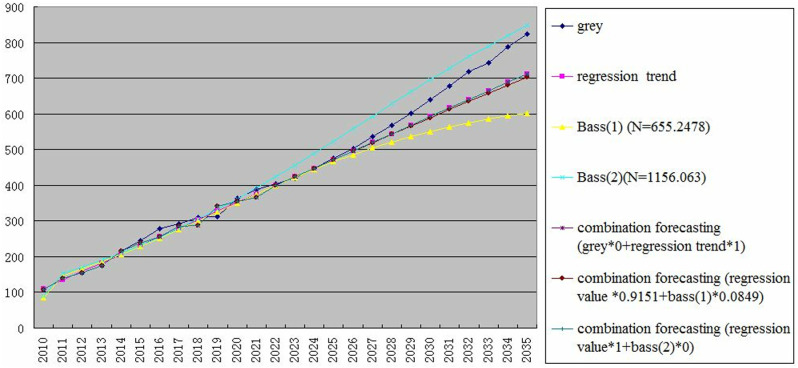
Summary of forecasting results.

We combine the above forecasting results into a graph, as shown in the following Fig 4.

In Fig 4, we summarize the forecasting results from the above various methods.

According to mean relative error (), the error percentages in grey forecasting method and regression trend method are respectively 5.373%, 2.9%. For the Bass model, the max error percentages is 5.94% in the forecasting of Bass (2) in Fig 4, but the lower error percentages of Bass model is 5.34% in the forecasting of Bass (1). Obviously, the used forecasting methods have different results, but the regression trend method has a better effect with the smallest error percentage of 2.9% in the paper. While the error percentages of grey forecasting and Bass model reach more than 5%, with a larger deviation. However, why should we continue to use combined forecasting? This is because regression trend forecasting has achieved good results in forecasting natural gas consumption in the logistics industry, but it cannot guarantee that it will also achieve good results in forecasting other energy consumption in the logistics industry, because there is an alternative energy in the logistics industry, and the trend of natural gas and electricity replacing gasoline and diesel is obvious. In order to provide more and better forecasting toolbox, we study and implement combined forecasting methods based on semi- hierarchical control. Surprisingly, the combined forecasting methods in this study have good forecasting stability, and the error is controlled in a small range, the error percentages of combined forecasting methods are within 2.9%−3.1%, the combined forecasting methods have also gotten better forecasting results.

In terms of computational reliability and complexity, we use the sum of error squares (as shown in formula Q above), and a reliable computer iterative method is also used to solve the problem. In combined forecasting method, the optimal solution of λ can be obtained according to this formula. It is surprising that the calculation complexity is not high, the scale is O(n), and n is the number of samples of the original sequence. Therefore, the reliability and complexity of the calculation in this study are good. For example, in the above single-method forecasting, the regression trend forecasting method has the best effect with minimum error and good reliability, while in the combined of grey forecasting and regression trend forecasting, λ is 1 according to the sum of error squares. This is equivalent to giving 100% weight to the regression trend forecasting method. Moreover, in the combined of regression trend forecasting and Bass model (1), λ is 0.9151 based on the sum of error squares, which is equivalent to giving 91.51% weight to the regression trend forecasting method. Therefore, The λ value obtained scientifically in the combined forecasting method is also of practical significance and operability, and has good reliability, and can control the forecasting error very well.

The results in Fig 4 show every forecasting method have their advantages and disadvantages. In the case of very optimistic demand for natural gas consumption in the logistics industry (such as the market demand for natural gas trucks and ships increases sharply), the forecasting values of the grey forecast method and the Bass model method (under the large ultimate carrying capacity) are more similar and more optimistic. In the case of medium optimism (cautiously optimism), the values of regression trend forecasting are good. If the natural gas consumption demand of the logistics industry in the future will not optimistic, the forecasting values of the Bass model (normal ultimate carrying capacity) can also reflect the natural gas consumption demand of the logistics industry. Admittedly, combined forecasting is a beneficial supplement, especially when the deviation values are too large between the forecasting values of a single method and the original actual values. We solve the problem of difficult determination of combined forecasting coefficients, and the combined forecasting methods have better forecasting stability.

Given China’s political stability and strong economic resilience, we prefer a scenario that is at least moderately optimistic (cautiously optimistic). With the further expansion of China’s natural gas exploitation, China’s self-sufficiency rate of natural gas consumption will gradually increase, which will be conducive to the steady decline of natural gas prices. In order to focus on high-quality development, The State Council officially issued the “Action Plan to promote large-scale equipment renewal and consumer goods for new” on March 13, 2024, which is likely to be a starting point, if the Chinese government implements administrative measures to adopt purchase subsidies for natural gas equipments or transportation vehicles in the future, we prefer a very optimistic scenario.

In general, there are the novelty points of this study comparing with previous methods.

(1) A semi-hierarchical control method is used in the process of natural gas forecasting in logistics industry. The top-down hierarchical control and inverse feedback mechanism for multi-method can ensure the error range to a very small degree.
(2) Multiple methods can be combined freely under semi-hierarchical control, the shortcomings of a single method can be avoided, especially in deviation and stability.
(3) The hard problem of freely combined forecasting coefficients under semi-hierarchical control is also solved, which can be calculated by the principle of minimum sum of error squares and can also be used conveniently for practical operation.

The novelty points are based on the use of inverse feedback mechanism of semi-hierarchical control, so the forecasting accuracy can be guaranteed.

The semi-hierarchical control method is available to expand the combined use of many forecasting methods, more forecasting models can be expectably added to the semi-hierarchical control method in the future.

## 5 Conclusions and policy implications

In the context of the “dual-carbon” strategy, natural gas acts as a clean energy, has been widely used in China’s logistics industry, especially in recent years, LNG began to be used in China’s logistics intercity transportation and port terminals. This paper takes these as starting points to study the current situation and trend forecasting of natural gas consumption in China’s logistics industry under semi-hierarchical control. Based on the above researches, the conclusions can be summarized as follows:

(1) In the analysis of the mechanism of gas- oil replacement, because natural gas is encouraged by China’s policies and the use cost of relatively cheap, the driving mechanism of natural gas instead of gasoline and diesel shows that gas instead of oil will exist for a long time. We believe that the logistics industry will continue to reduce its dependence on gasoline and diesel. Natural gas is a clean energy, China encourages the promotion of natural gas vehicles, and natural gas-powered heavy trucks and ships can better replace gasoline and diesel heavy trucks and transport vessels. From the perspective of the consumption trend of natural gas in the logistics industry, its economy and energy saving and low carbon are conducive to promoting the process of gas substitution for oil in China’s logistics industry.
(2) From the perspective of consumption forecasting, Bass model is a more flexible with the help of parameter adjustment. The forecasting results of grey forecasting and Bass model under large limit carrying capacity are optimistic, and the regression trend forecasting results are cautiously optimistic. Even if the assessment is not optimistic, the forecasting values of Bass model (normal carrying capacity) can also reflect the future gas consumption demand of the logistics industry. The forecast results in each method show that the natural gas consumption in the logistics industry will get a rapid growth and its demand will remain at a high level in the future for a long time. Furthermore, the forecasting results under different circumstances are helpful for government departments to estimate the consumption trend of natural gas in the logistics industry according to different market environments.
(3) Based on the reverse feedback mechanism of semi- hierarchical control, some combined forecasting methods are established. By adopting the principle of minimum sum of error squares, one problem that the combined forecasting coefficient is difficult to determine is solved, another problem that a single forecasting method is difficult to meet the forecasting of natural gas consumption in the logistics industry is also solved, these will further improve the forecasting accuracy of natural gas consumption in the logistics industry.

This study has obtained some valuable insights and results, as evident from the above research. In addition, there are few literatures on the forecasting of natural gas consumption in the logistics industry at home and abroad, so this study has good reference value. In the future, with the demand expansion of natural gas consumption, it will further promote the development of green and low-carbon logistics. Through this study, suggestions for Chinese government are as follows:

(1) Strengthen natural gas exploitation and reduce external dependence. In a natural gas trade deficit environment in China, China needs to strengthen natural gas exploitation. It can vigorously develop the “coal to gas” project and natural gas distributed energy stations, and vigorously promote the applications of natural gas in civil, transport and storage industries.
(2) Accelerate the process of natural gas price reform, and vigorously attract private capital to participate in natural gas exploration, storage and transportation, equipment manufacturing, and gas station construction. About natural gas pricing reform and marketization in China, there existed many discussions, just like the study of Liu [1], reforms can be piloted in multiple cities and then transmitted nationwide, China can effectively enhance natural gas market vitality by speeding up price reform. Moreover, Private capital is needed to participate in natural gas industry chain, because the efficiency of private capital is higher, and if it enters the natural gas energy industry chain on a large scale, it will effectively promote the development of the natural gas industry chain, and then be transmitted and benefited to the logistics industry.
(3) Accelerate the promotion and application of trucks and transport vessels powered by natural gas, increase the use of natural gas energy-saving equipment in the logistics industry, and increase the proportion of natural gas consumption in the energy consumption structure of the logistics industry, which can promote the development of the logistics industry towards a clean and low-carbon direction, so as to make greater contributions to China’s “dual-carbon” strategy.

## References

[1] Liu SY. Research on supervision and examination on pricing cost of China’s urban pipeline natural gas – taken Shenyang as example. Prices Monthly. 2018;2:18–22. doi: 10.14076/j.issn.1006-2025.2018.02.04

[2] Li TD, He CL, Dong ZY, Zhang Y. Paths and policies to perfect pricing mechanism for China natural gas. Natural Gas Technology and Economy. 2021;15(1):68–75. doi: 10.3969/j.issn.2095-1132.2021.01.011

[3] Chen R, Qi PF, Zhang XY, Li CX. Evolution laws and new trends of global LNG pricing and their implications. Natural Gas Industry. 2021;41(05):144–52. doi: 10.3787/j.issn.1000-0976.2021.05.016

[4] Xie RL. European natural gas pricing and enlightenment for Chinese natural gas market. Chemical industry. 2023;41(3):27–35. doi: 10.3969/j.issn.1673-9647.2023.03.007

[5] Apergis N, Payne JE. Natural gas consumption and economic growth: A panel investigation of 67 countries. Applied Energy. 2010;87(8):2759–63. doi: 10.1016/j.apenergy.2010.01.002

[6] Bianco V, Scarpa F, Tagliafico LA. Analysis and future outlook of natural gas consumption in the Italian residential sector. Energy Conversion and Management. 2014;87:754–64. doi: 10.1016/j.enconman.2014.07.081

[7] Madreimov T. The Influence of Gas Consumption and Extraction for Economic Growth: a 53 Gas Producing Country Empirical Assessment. Doctoral dissertation from China University of Petroleum (East China); 2019. p. 1–31.

[8] Dong KY, Sun RJ, Li H. Study on the adjustment and countermeasures of natural gas consumption structure in China. Science and Technology Management Research. 2016;9:235–41. doi: 10.3969/j.issn.1000-7695.2016.09.043

[9] Li QC, Zhang P, Chen JH. Natural-gas industry in Sichuan province: A coupling degree of security-economy environmental protection from the perspective of energy “trilemma”. Natural Gas Technology and Economy. 2024;18(01):7–14. doi: 10.3969/j.issn.2095-1132.2024.01.002

[10] Soldo B. Forecasting natural gas consumption. Applied Energy. 2012;92:26–37. doi: 10.1016/j.apenergy.2011.11.003

[11] Majazi Dalfard V, Nazari Asli M, Asadzadeh SM, Sajjadi SM, Nazari-Shirkouhi A. A mathematical modeling for incorporating energy price hikes into total natural gas consumption forecasting. Applied Mathematical Modelling. 2013;37(8):5664–79. doi: 10.1016/j.apm.2012.11.012

[12] Baldacci L, Golfarelli M, Lombardi D, Sami F. Natural gas consumption forecasting for anomaly detection. Expert Systems with Applications. 2016;62:190–201. doi: 10.1016/j.eswa.2016.06.013

[13] Zhang W, Yang J. Forecasting natural gas consumption in China by Bayesian Model Averaging. Energy Reports. 2015;1:216–20. doi: 10.1016/j.egyr.2015.11.001

[14] Zou LM, Tang YX. Prediction of China’s natural gas import volume and transportation safety evaluation based on machine learning. Journal of Industrial Technological Economics. 2025;2:108–18. doi: 10.3969/j.issn.1004-910X.2025.02.011

[15] Zhang MQ. The impact mechanism and trend prediction of China’s natural gas demand under the background of dual carbon goals. Economic Forum. 2024;6:71–87.

[16] Zheng CD, Lan YN. Natural Gas Demand Forecasting Based on XGboost and Prophet Combined Model. Urban Gas. 2024;12:32–6. doi: 10.3969/j.issn.1671-5152.2024.12.007

[17] Wen Q, Wang N, Wei XH. Natural Gas Demand Forecast Model Based on MF-DFA and BorutaShap. Oil & Gas Storage and Transportation. 2025;44(1):109–19. doi: 10.6047/j.issn.1000-8241.2025.01.011

[18] Zhen Q. Decomposition of natural gas consumption structure in typical countries and China’s consumption forecast. China University of Geosciences (Beijing); 2020. p. 1–40.

[19] Li T, Ma X, Wu W, He Q. Research on novel nonlinear Bernoulli grey model with hybrid accumulation and its application in forecasting natural gas production and consumption. Communications in Nonlinear Science and Numerical Simulation. 2025;146:108773. doi: 10.1016/j.cnsns.2025.108773

[20] Qin F, Tong M, Huang Y, Zhang Y. Modeling, prediction and analysis of natural gas consumption in China using a novel dynamic nonlinear multivariable grey delay model. Energy. 2024;305:132105. doi: 10.1016/j.energy.2024.132105

[21] Qiao W, Ma Q, Yang Y, Xi H, Huang N, Yang X, et al. Natural gas consumption forecasting using a novel two-stage model based on improved sparrow search algorithm. Journal of Pipeline Science and Engineering. 2025;5(1):100220. doi: 10.1016/j.jpse.2024.100220

[22] Xu X, Xu H, Mei K, Tong L, Liu Z, Wang T, et al. Integrating multi-modal data into transformer model for short-term gas consumption forecasting. Alexandria Engineering Journal. 2025;122:655–64. doi: 10.1016/j.aej.2025.03.029

[23] Zhang J, Shen C, Qin Y, Chen J. Forecasting of natural gas based on a novel discrete grey seasonal prediction model with a time power term. Energy Strategy Reviews. 2025;58:101677. doi: 10.1016/j.esr.2025.101677

[24] Ma X, Deng Y, Ma M. A novel kernel ridge grey system model with generalized Morlet wavelet and its application in forecasting natural gas production and consumption. Energy. 2024;287:129630. doi: 10.1016/j.energy.2023.129630

[25] Wang Y, Fan N, Wen S, Kuang W, Yang Z, Xiao W, et al. A novel structural adaptive discrete grey Euler model and its application in clean energy production and consumption. Energy. 2025;323:135807. doi: 10.1016/j.energy.2025.135807

[26] Li H, Duan H, Song Y, Wang X. A novel conformable fractional logistic grey model and its application to natural gas and electricity consumption in China. Renewable Energy. 2025;243:122591. doi: 10.1016/j.renene.2025.122591

[27] Zhao Y, Yang Y, Jia R, Chai J. Analysis of the rebound effect and induced effect of non-residential natural gas consumption: Empirical evidence from China. Journal of Management Science and Engineering. 2025;10(1):54–67. doi: 10.1016/j.jmse.2024.08.002

[28] Chen F, Wang FZ, Li CY. Research on energy price, energy substitution and low carbon logistics development: Taking Zhejiang Province as an example. Price Theory & Practice. 2019;2:145–8. doi: 10.19851/j.cnki.cn11-1010/f.2019.02.039

[29] Zhao J. Preparation storage and transportation of liquefied natural gas (LNG). Modern Chemical Research. 2023;24:174–6. doi: 10.20087/j.cnki.1672-8114.2023.24.056

[30] Lei H, Guo Y, Khan N. Forecasting energy use and efficiency in transportation: Predictive scenarios from ANN models. International Journal of Hydrogen Energy. 2025;106:1373–84. doi: 10.1016/j.ijhydene.2025.01.474

[31] Micallef A, Apap M, Licari J, Spiteri Staines C, Xiao Z. A comparative framework for evaluating machine learning models in forecasting electricity demand for port microgrids. Energy and AI. 2025;20:100494. doi: 10.1016/j.egyai.2025.100494

[32] Sun C, Cao L, Lei FY. Design of hierarchical intelligent control system for facility agricultural logistics based on AGV. Rural Science and Technology. 2023;14(7):155–8. doi: 10.19345/j.cnki.1674-7909.2023.07.015

[33] Hou S, Chen H, Liu X, Cui J, Zhao J, Gao J. Hierarchical model predictive control for energy management and lifespan protection in fuel cell electric vehicles. Energy. 2025;319:134968. doi: 10.1016/j.energy.2025.134968

[34] Chen X, Hu Y, Zhao J, Chen Z, Li Z, Yang H. Real-time optimal dispatch for large-scale clean energy bases via hierarchical distributed model predictive control. Applied Energy. 2025;385:125503. doi: 10.1016/j.apenergy.2025.125503

[35] Wang T. System engineering. Chongqing University Press; 2020.

[36] Wang BH, Wang S. Python Data Analysis and Basic Course. Publishing House of Electronics Industry; 2018.

[37] Zhu Y, Tokimatsu K, Matsumoto M. A Diffusion Model for Natural Gas Vehicle: A Case study in Japan. Energy Procedia. 2015;75:2987–92. doi: 10.1016/j.egypro.2015.07.607

[38] Fujita M, Krugman P, Venables AJ. The spatial economy: Cities, regions and international trade. China People’s Publishing House; 2005.

